# A Whole-Genome DNA Marker Map for Cotton Based on the D-Genome Sequence of *Gossypium raimondii* L.

**DOI:** 10.1534/g3.113.006890

**Published:** 2013-10-01

**Authors:** Zining Wang, Dong Zhang, Xiyin Wang, Xu Tan, Hui Guo, Andrew H. Paterson

**Affiliations:** Plant Genome Mapping Laboratory, University of Georgia, Athens, Georgia 30605

**Keywords:** quantitative trait loci, resistance gene analog, simple sequence repeat, restriction fragment length polymorphism, inversions

## Abstract

We constructed a very-high-density, whole-genome marker map (WGMM) for cotton by using 18,597 DNA markers corresponding to 48,958 loci that were aligned to both a consensus genetic map and a reference genome sequence. The WGMM has a density of one locus per 15.6 kb, or an average of 1.3 loci per gene. The WGMM was anchored by the use of colinear markers to a detailed genetic map, providing recombinational information. Mapped markers occurred at relatively greater physical densities in distal chromosomal regions and lower physical densities in the central regions, with all 1 Mb bins having at least nine markers. Hotspots for quantitative trait loci and resistance gene analog clusters were aligned to the map and DNA markers identified for targeting of these regions of high practical importance. Based on the cotton D genome reference sequence, the locations of chromosome structural rearrangements plotted on the map facilitate its translation to other Gossypium genome types. The WGMM is a versatile genetic map for marker assisted breeding, fine mapping and cloning of genes and quantitative trait loci, developing new genetic markers and maps, genome-wide association mapping, and genome evolution studies.

Genetic mapping is an essential prerequisite for the activities of marker assisted selection, gene/quantitative trait loci (QTL) cloning, genome sequence assembly, association mapping, and evolutionary studies (Duran *et al.* 2009). Genetic marker systems such as restriction fragment length polymorphism (RFLP), simple sequence repeats (SSR), sequence-related amplified polymorphism, and others have been widely used in linkage and QTL mapping (Agarwal *et al.* 2008) in population sizes of up to a few hundred individuals, generally involving highly divergent parents and in strong linkage disequilibrium such that a few hundred markers provided adequate information. Whole-genome genotyping methods that are now being reduced to routine and cost-effective practice (Xie *et al.* 2010; Andolfatto *et al.* 2011; Elshire *et al.* 2011; Bus *et al.* 2012) open the door to investigations such as global trait mapping and association that require vastly greater DNA marker densities (Poland *et al.* 2011) and are likely to render many previous marker systems obsolete. However, the mapping information garnered from previous systems remains valuable, with individual QTL and particularly meta-analyses (Rong *et al.* 2007; Zhang *et al.* 2013) identifying genomic regions that might be searched at high resolution for causal genes.

High-contiguity “reference” genome sequences provide a natural platform for unifying information from a range of sequence-tagged DNA marker systems toward the efficient application of new approaches to build upon collective knowledge of the biology of an organism. Most major crops now have a reference genome sequence, and some authors have projected that within a few years all of the ~200 widely used domesticates will have such a resource (Paterson 2006). For the organism that we focus on herein, cotton (Gossypium), the smallest of eight genome types (A−G, and K) and a suspected progenitor of cultivated polyploids, the D-genome of *Gossypium raimondii*, recently was sequenced (Paterson *et al.* 2012; Wang *et al.* 2012). A high degree of colinearity between various genomes in the Gossypium genus (Reinisch *et al.* 1994; Brubaker *et al.* 1999; Han *et al.* 2004; Rong *et al.* 2004; Desai *et al.* 2006; Becerra Lopez-Lavalle *et al.* 2011) suggests that the D-reference genome will extrapolate well to most regions of most other Gossypium genomes.

In cotton, more than 30 genetic maps have been published, largely based on interspecific crosses between two species that are each domesticated but are treated by breeders as different gene pools, *G. hirsutum* × *G. barbadense* (Jiang *et al.* 1998; Zhang *et al.* 2002; Lacape *et al.* 2003; Nguyen *et al.* 2004; Rong *et al.* 2004; Guo *et al.* 2007; He *et al.* 2007; Yu *et al.* 2007; Lacape *et al.* 2009). The interspecific tetraploid genetic maps were valuable for finding new DNA markers at a time that primary genetic mapping was a high priority (Reinisch *et al.* 1994; Nguyen *et al.* 2004; Rong *et al.* 2004; Guo *et al.* 2007; Zhang *et al.* 2008; Yu *et al.* 2011). However, conventional breeding programs seldom use such wide crosses (Ulloa and Meredith 2000; Ulloa *et al.* 2002)—and intraspecific crosses, largely within *G. hirsutum* (‘Upland’ cotton) suffered from a paucity of DNA polymorphism that often left much of the genome unmapped (Shappley *et al.* 1998a, b; Ulloa and Meredith 2000; Ulloa *et al.* 2002; Rong *et al.* 2005a; Shen *et al.* 2005, 2007; Wang *et al.* 2007; Chen *et al.* 2008; Xu *et al.* 2008; Zheng *et al.* 2008; Liu *et al.* 2011).

Increasing the DNA marker density of cotton genetic maps is urgent for marker-assisted selection and genomic studies. This need has been widely recognized, and several efforts have interleaved existing maps based on subsets of shared DNA markers to form “consensus” (Rong *et al.* 2005b) or “integrated” maps (Yu *et al.* 2010; Reddy *et al.* 2011; Blenda *et al.* 2012) with as many as 8254 loci (Blenda *et al.* 2012).

In this study, we constructed a whole-genome marker map (WGMM) by integrating publicly available sequence tagged DNA markers with the cotton D-genome sequence. Chromosomal affiliations were deduced based on DNA markers derived from diploid and tetraploid cotton genetic maps (Rong *et al.* 2004), and we noted known differences among the genomes and subgenomes that should be considered when the cotton markers and maps are used. ‘Hotspots’ for QTL previously implicated in fiber development, and clusters of resistance-gene analogs (RGAs) identified in the genome sequence, also were aligned with the WGMM. The WGMM provides a foundational tool and resources for increasing knowledge of fundamental and applied elements of cotton biology, via marker-assisted breeding, fine mapping and cloning genes and QTL, genome wide association mapping, and other studies.

## Materials and Methods

The materials used in this study include the D-genome sequence for *G. raimondii* (Paterson *et al.* 2012), a consensus genetic map and diploid D-genome genetic map (Rong *et al.* 2005a), tetraploid At and Dt maps (Rong *et al.* 2004; 2005a), a cotton QTL meta-analysis (Rong *et al.* 2007), and the Cotton Marker Database (http://www.cottonmarker.org/).

### Construction of WGMM

The availability of sequence-tagged markers such as RFLP probes and SSR in the Cotton Marker Database (http://www.cottonmarker.org/) and a cotton consensus map (Rong *et al.* 2005a) provides alignable information to convert genetic positions (in centiMorgans, cM) of markers to physical positions (bp).

After marker sequences were prepared, Blastn (Altschul *et al.* 1990) was applied to anchor markers to the cotton D-genome pseudo molecules. Markers with alignments of E-value ≤ 1e-10 for RFLP/SSR sequences and ≤50 for SSR primers were assembled into loci. For RFLPs, the alignments with distance ≤5000 bp were assembled into one RFLP locus. For SSR primers, one forward primer hit was combined with one reverse primer hit if the distance between the two hits was ≤1000 bp.

Colinearity between genetic and physical positions was determined by ColinearScan 1.0.1 (Wang *et al.* 2006). The colinear markers aligned on the D-genome molecules were maintained as anchoring markers with their original genetic distance. The genetic distances of the noncolinear markers were estimated based on the genetic distance between the anchoring markers and the physical locations of the markers. Strikingly discrepant loci were removed, based on the order of markers in the original source. A QTL region was delineated by two flanking markers nearest to the likelihood peak that had alignment information.

### Identification of RGA clusters

All cotton proteins were used to search for nucleotide binding site (NBS) domains (PF00931, NB-ARC) by a Hidden Markov Model method (Eddy 1998) implemented in hmmsearch version 3.0 with e-value cutoff = 1. To filter false-positive hits, all identified NBS containing proteins were screened against the Pfam-A file (Bateman *et al.* 2004). NBS domains that overlap with other domains with lower E-value were considered false hits and abandoned. Likewise, the Toll/Interleukin-1 receptor (TIR) domain (PF01582) was searched against all cotton proteins by hmmsearch with e-value cutoff = 1 and putatively false hits abandoned. To detect LRR motifs, predicted NBS encoding proteins were searched against 10 LRR families in the LRR clan (CL0022) with e-value cutoff = 1. All regions predicted as LRR motifs and not overlapping with other domains with lower e-value were inferred to be real LRR motifs. Coiled coil (CC) motifs were detected by the use of NCOILS software (http://bioserv.cbs.cnrs.fr/htbin-post/pat/new/wpat.pl?dir=example_1&tool=ncoils) with default parameters. CCs at the N-terminus of NBS domains and not overlapping with other domains were considered to identify CC-NBS type genes. Only the RGA clusters (clusters containing only RGAs) were selected for this study.

### Dotplots

To compare the genetic maps constructed for diploid D, tetraploid At and Dt, and a consensus map (Rong *et al.* 2004, 2005a), the markers in these maps were mapped onto the cotton pseudomolecules by running Blastn and using their sequences against the constructed cotton assembly at the criteria of e-value < 1e-10. Dotplots of genetic maps and the pseudomolecules were generated with an in-house perl program (similar to those available at http://chibba.agtec.uga.edu/duplication/).

## Results

### Marker number and density on chromosomes

The total of 20,096 sequence-tagged cotton DNA markers were first filtered for sequence/primer duplications. In total, 18,597 nonredundant markers (Table 1) were used to do Blastn searches against the D-genome pseudomolecules, and 79,481 sequence alignments could be aligned to the D genome. After we filtered clustered duplicate copies within 1 kb for SSR and 5 kb for RFLP, 48,546 loci were mapped on the 13 chromosomes of WGMM and 412 loci on the unassembled scaffolds (Supporting Information, Table S1). The 18,597 markers used for the alignments have an average of 2.63 copies per marker sequence, ranging from 6493 (34.91%) markers with a single copy to 1195 markers with four copies (6.43%: Table 1). Among the 48,958 mapped loci, 42,794 (87.41%) are SSRs and 6164 (12.59%) are RFLPs. Loci from markers with five copies accounted for the largest share (45.81%) of the 48,958 loci, and the loci from four copy-markers had the smallest share (9.76%).

**Table 1:** Numbers and percentages of markers and loci on the WGMM

No. Copy	No. Markers	%	No. Loci	%
1	6,493	34.91	6,493	13.26
2	4,012	21.57	8,024	16.39
3	2,412	12.97	7,236	14.78
4	1,195	6.43	4,780	9.76
5	4,485	24.12	22,425	45.81
Total	18,597	100	48,958	100

WGMM, whole-genome marker map.

Marker density was closely correlated with the physical DNA content of chromosomes (r = 0.81; *P* = 0.0004), with size differences among the 13 chromosomes reflected by differences in locus number (Table S2). D12, with 34.9 Mb DNA, has the fewest loci mapped (2386); D09 with 70.7 Mb has the most (5370). Marker density along a chromosome ranges from 12.8 to 18.5 kb per locus (D07, D10), with an average of 15.6 kb per locus. Relative to the 37,505 genes in the current D genome annotation, the WGMM provides 1.3 marker loci per gene.

### Marker distribution on chromosomes

**Figure 1:**
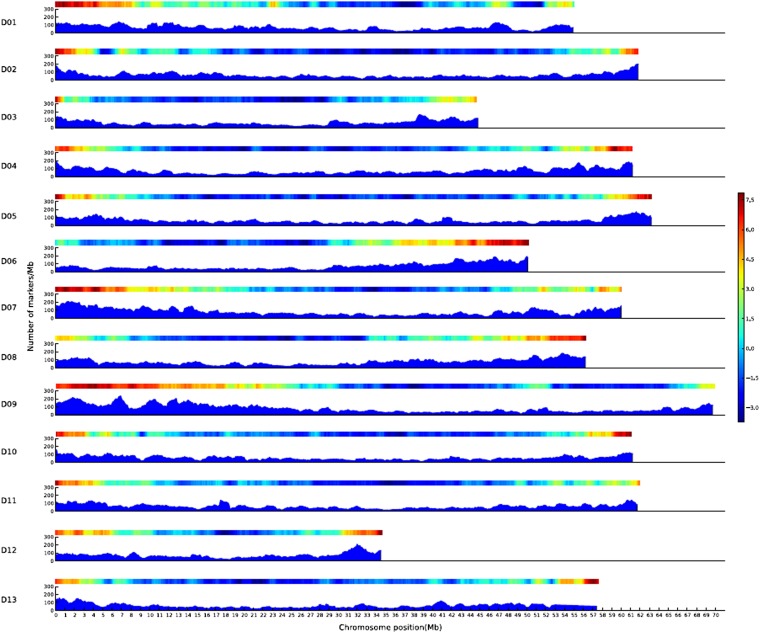
DNA marker, gene, and repetitive DNA distributions on the 13 D-genome pseudomolecules and the WGMM. The x-axis indicates the lengths of the chromosomes in Mb and the y-axis shows the number of markers per 1 Mb window in a range of 0−300. The shaded fields in blue display the marker distribution in 1-Mb windows at 100-kb steps from 0 Mb to the end of each chromosome. Gene (red) and repetitive sequence (blue) density heatmaps for the chromosomes are above each marker distribution map.

With one exception, the cotton chromosomes have relatively greater marker densities of 100−220 markers per 1 Mb in terminal chromosomal regions that are also gene-rich (Figure 1), and significantly lower marker densities (50−120 loci per Mb) in central regions that are gene-poor and repeat-rich (see the gene and repeat density heatmap in Figure 1). A few greater peaks appear in the middle regions of some chromosomes that are also gene rich.

To assess the coverage of this map, we determined the number of markers in each of the 34−69 1-Mb windows of D01-D13. The least-populated window (on D02) had nine markers and a physical distance of 110 kb between markers, indicative of good coverage of the whole genome by these markers. The WGMM covered 98.11% of the 747 Mb assembled portion of the cotton D genome of total 761.4 Mb (Paterson *et al.* 2012).

One unusual chromosome, D06, had high marker density consistent with the remaining chromosomes at one end but unusually low density at the other end that was more consistent with the central regions of other chromosomes (Figure 1). The unusual chromosome arm (0−27 Mb) had lower gene and higher repeat densities that may have been the main reasons for the lower marker density, noting that most markers were SSRs developed from expressed sequence tags. We also searched the ribosomal DNA (rDNA) sequences. The rDNA (18S, 5S, and 25S) was mainly distributed on three chromosomes, with 40 rDNA genes in a 249-kb region (20,789,590−21,038,157 bp) and a 544-kb region (32,723,102−33,266,822 bp) on D08, 45 rDNA genes in a 400-kb region (43,290,160−43,692,820 bp) on D09 and 111 rDNA genes in a 362-kb region (55,503,532−55,865,427 bp) on D01 (Figure 1). Another 33 rDNA genes were scattered, with 1−11 gene copies on D02, 03, 04, 05, 06, 07, 10, 12, and 13. The rDNA genes caused lower marker density in local regions that were too short to be labeled in the figure and did not significantly influence marker and gene density.

### Marker alignment from consensus map to WGMM

To integrate recombination patterns into the WGMM, it was aligned with a consensus genetic map of 2325 cM (Table S2) that was used in assembling the D genome pseudo molecules, and has been confirmed to be a good representation of At and Dt genetic maps (Table S1; Table S2; Rong *et al.* 2004). Altogether, 1725 markers from the consensus map could be mapped on the WGMM, with 86 to 220 per chromosome (Table S3). Approximately 60% of the mapped markers showing colinearity between physical and genetic locations on the D genome were used as anchor markers on WGMM and their genetic distances transferred to the WGMM. Genetic locations of nonanchor markers on the WGMM were interpolated by using the genetic and physical spacings of flanking markers.

### Cotton fiber QTL hotspots

From a meta-analysis of 432 cotton QTL (Rong *et al.* 2007), we mapped to the WGMM 104 fiber QTL that comprised 18 “hotspots” containing QTL for 3−7 fiber traits including fiber elongation, color, fineness, length, strength, uniformity, micronaire and short fiber content (Table 2). Chromosome 07 and 23 each have the largest number of 16 QTL. Table 2 lists the interval locations and the number of markers in the intervals on D-genome molecules.

**Table 2:** QTL from At and Dt tetraploid chromosomes with their consensus locations and markers on the D genome and WGMM

At & Dt Chr.	No. QTL	D Chr.	Start bp	End bp	No. Markers	QTL traits	QTL names
7;16	4	D01	17,773,028	45,685,292	1,433	FF;FL;FU;MIC	FU16.1(FLU);FL16.1;FF16.1;MIC16.1
1;15	5-6	D02	1,390,547	20,704,735	1,344	EL;FF;FL;FS;FU;MIC	FL01.1(HVsl2.5,HVuhm);EL01.1;FU01.1(HVlnCV);FL01.2(HVsl2.5);FS01.2;EL01.1;FF01.1;MIC01.1;FF01.2;EL01.2;FF15.1
2/3;17	5	D03	42,011,485	45,719,191	386	FC;FL;FU;MIC	FL17.1(HVsl2.5,HVuhm);FU17.1(HVur);MIC17.1;FS17.1;FC17.1
2/3;17	5	D03	3,185,870	35,433,985	1,387	FL;FS;FU;SF	FL03.1(HVuhm,HVsl2.5);FU03.1(HVur);SF03.1(SFCn);FS02.1;FS02.2
LGA02;LGD03	4-5	D04	50,443,620	58,823,186	803	EL;FF;FL;FU	FFD03.05;FFD03.1;FLD03.1(HVsl2.5,HVuhm,Lw);FUD03.1(HVui);ELD03.1(ELONG)
2/3;14	5-6	D05	39,041,081	62,380,583	1,366	FF;FL;FS;FU;MIC	FF14.05;FU14.2(FLU);FL14.1(HVuhm);MIC14.2;FF14.1;FS14.1
2/3;14	6-7	D05	4,002,919	49,222,277	2,191	EL;FF;FL;FU;MIC	EL02.1;FF02.1;FL02.1(HVsl2.5,HVuhm,Lw);FU02.1(HVui);MIC02.1;FL02.2(HVuhm);FF02.2;FF03.1
9;23	4-6	D06	37,014,370	47,605,556	1,238	EL;FF;FS;FU	EL23.03;FF23.1;EL23.05(FE);EL23.1;FU23.1(HVui);FS23.2;FS23.1
LGA03;LGD02	4-6	D07	17,026,813	48,701,291	1,541	EL;FC;FF;FL;FS	FFD02.1;FLD02.2(HVsl2.5);FSD02.1;FSD02.2(STR);FCD02.1;ELD02.1
LGA03;LGD02	6	D07	13,773,747	19,329,003	485	EL;FF;FL;FS;FU	ELA03.1(HVsl2.5,HVuhm,Lw);FLA03.1;FFA03.2;FSA03.1;FLA03.2;FUA03.2(FLU)
LGA03;LGD02	4-5	D07	28,919,943	57,194,167	1,455	EL;FF;FL;FU	FUA03.1(HVui);ELA03.1(HVsl2.5,HVuhm,Lw);FLA03.1;FFA03.1;FFA03.2
LGA03;LGD02	5	D07	3,099,961	5,211,929	328	EL;FC;FL;FU	FLA03.2;FUA03.2(FLU);ELA03.2;FUA03.3(HVui);FCA03.1
12;26	4-6	D08	37,154,657	55,710,457	1,812	EL;FF;FL;FS;FU;MIC;SF	FL26.1(HVsl2.5,HVuhm,Lw);FL26.2(HVuhm);EL26.1;MIC26.1;FF26.1;MIC26.2;FS26.1;FU26.1(FLU);FS26.2(STR);FS26.3;SF26.2;FU26.2(HVui)
12;26	4-6	D08	41,534,671	55,710,457	1,450	EL;FF;FL;FU	FF12.1;FF12.15;FL12.1(HVsl2.5,HVuhm);FU12.2(HVlnCV,HVur);EL12.1;FF12.2
4/5;LGD08	9	D09	4,942,098	9,480,129	552	EL;FF;FL;FS;FU;MIC;SF	ELD08.1;FFD08.1(HVsl2.5,HVuhm,Lw);MICD08.1;ELD08.2;FLD08.1(HVsl2.5,HVuhm,Lw);FUD08.1(HVui);SFD08.1(HVsfc,SFCn);FSD08.2(STR);FFD08.2
4/5;LGD08	5-7	D09	12,049,737	29,728,227	1,773	EL;FF;FL;FU;MIC;SF	FFD08.3;MICD08.2;ELD08.3;ELD08.4;FLD08.2(HVsl2.5,HVuhm,Lw);FUD08.2(HVui);SFD08.2(HVsfc);SFD08.15;FFD08.4;MICD08.3
4/5;LGD08	5	D09	11,863,128	17,291,302	750	EL;FF;MIC	FF05.15;MIC05.1;FF05.2;EL05.05(FE);FF05.3
10;20	5	D11	2,519,220	3,161,683	57	EL;FF;FL;SF	EL20.1;FL20.1(HVsl2.5,Lw);SF20.1(SFCn);FL20.15(HVuhm);FF20.1

QTL, quantitative trait loci; WGMM, whole-genome marker map; EL: elongation; FC: color; FF: fineness; FL: length; FS: strength; FU: uniformity; MIC: micronaire; SF: short fiber content

### Cotton RGA hotspots

A total of 63 RGA clusters were identified on 9 of the 13 D-genome chromosomes (Table S4). D07 has the largest number of clusters (21), whereas D04 has the smallest (one, with only two RGAs). The cluster positioned at 51,589,128 bp on D09 is the largest cluster with seven RGAs. Because there is no other gene in the RGA clusters, some clusters with only two RGAs have no markers within the interval whereas the largest cluster has five markers. Cluster positions and their closest flanking markers are listed (Table S4) to facilitate the use of these markers in expedited searches for loci responsible for disease and/or pest resistance phenotypes.

### Comparative map alignments of Di, At, Dt to the D-genome sequence

To further enhance alignment of the WGMM to previous genetic information, we aligned the genetic maps of the diploid D (Rong *et al.* 2004), tetraploid At and Dt (Reinisch *et al.* 1994; Lacape *et al.* 2003; Rong *et al.* 2004), with the D-genome pseudo molecules. To avoid confusion, Di represents the D genome genetic map and D the D-genome sequence in the text, tables, and figures hereafter. All chromosomes from each genetic map could be aligned to the WGMM with marker numbers per chromosome of 21−65 (Di), 20−93 (At), and 38−95 (Dt). The numbers of aligned markers on the corresponding and other chromosomes are listed in Table S3.

### Genome variations

**Figure 2:**
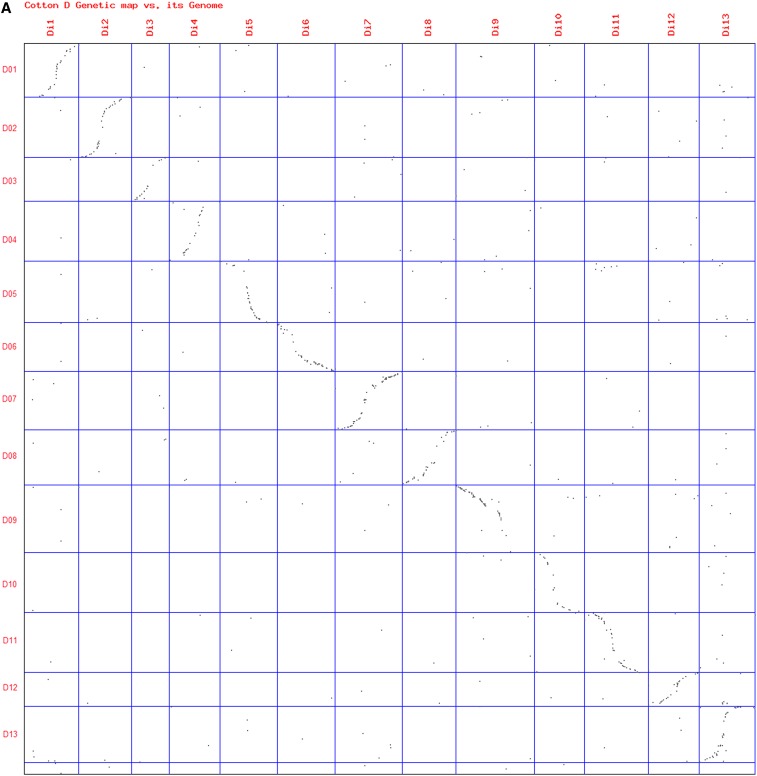

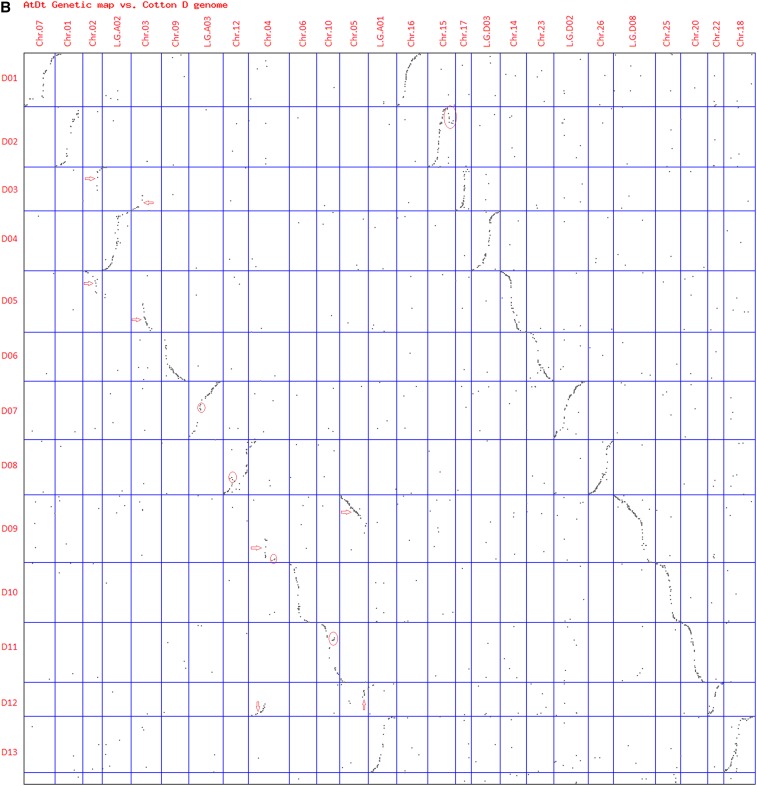
Comparison of the Di, At, and Dt genetic maps and their physical locations on the pseudo molecules of the D-genome sequence of *G. raimondii*. The 13 D-genome pseudo molecules are plotted on all y-axes. (A) Di01-Di13 indicates the 13 chromosomes on the D-genome genetic map; (B) Chr.01−Chr.26 indicates the 26 tetraploid chromosomes of cotton. Both the relative genetic and physical distances of the chromosomes on the plots are represented by the cells of different sizes according to the ratio of the chromosome lengths. Chromosome arm inversions (red ovals) and translocations (red arrows) are highlighted.

To study the colinearity and genome variations between the genetic maps and the D genome sequence, dotplots were performed between the Di, At, and Dt genetic maps and the D genome pseudomolecules (Figure 2, A and B). Here, we listed the large inversions (>1 Mb) different from those found in Rong *et al.* (2004) based on the comparison of genetic maps. The D genome diploid genetic map had been used to construct the pseudomolecules, and therefore, the dotplot between them showed good consistency as expected (Figure 2A). The Dt genetic map also exhibited colinearity with 11 of the pseudo molecules, with chromosome 15 having an inversion (Figure 2B, red oval) of 14.71 Mb accounting for 23.44% of D02 (Table 3). Four inversions (Figure 2B) relative to At chromosomes with sizes ranging from 3.33 to 6.27 Mb were identified on Chromosomes 04, 10, 12, and LGA03, accounting for 5.36–9.95% of the corresponding chromosomes.

**Table 3:** Genome variations identified via comparative mapping against D-genome pseudomolecules

Chromosome	Genetic Interval, cM	cM	Total cM	% of chr.	Start Marker	End Marker	Physical interval, Mb	Mb	Chr. Length, Mb	% of Chr.	Events
Chr.15-D02	169.9−123.2	46.7	176.4	26.47	PAR08C07	PAR0935	17,037,231-2,325,639	14.71	62.75	23.44	Inversion
LGA03-D07	69.4−84.4	15	198	7.57	Gate4DB08	Gate1BF07	28,319,670-32,633,603	4.31	60.74	7.09	Inversion
Chr.12-D08	55.4−59.0	3.6	213.6	1.69	ESTS146	Coau2I13	39,347,694-43,302,432	4.00	58.26	6.87	Inversion
Chr.04-D09	166.7−181.8	15.1	186.1	8.11	PAR0372	Gate3DC07	66,859,322-70,184,607	3.33	62.13	5.36	Inversion
Chr.10-D11	111.2−85.2	26	182.7	14.23	BNL1161	pVNC163	16,252,771-22,482,522	6.23	62.59	9.95	Inversion

Chr., chromosome.

The At genomes displayed two reciprocal chromosome arm translocations (Rong *et al.* 2004) between chromosome 02/03 and between chromosome 04/05 (red arrows in Figure 2B) and their physical location information on the D-genome sequence was added here. The translocations broke the chromosomes near centromere regions, such as chromosome 02/03 corresponding to pseudo molecule D03 at 2,4043,418−29,931,543 bp (PAR0499−A1171) and chromosome 03/02 to D05 at 23,203,279−3,4341,901 bp (A1325-Unig26F10); chromosome 04/05 to D09 at 46,366,195−39,825,818 bp (Unig27B06-Gate4DF07) and chromosome 05/04 to D12 at 2,1985,484−24,460,316 bp (Unig06C12-Coau2105). Table 3 lists the flanking markers for the five inversions with their locations on both the genetic maps and the WGMM.

## Discussion

The cotton WGMM described herein has a total of 48,959 loci, about six times the number characterized in the most richly populated of the integrated maps published previously (Blenda *et al.* 2012). Comparable with a linkage map of rice based on population sequencing with 15,795 SNPs (Xie *et al.* 2010) and a Brassica genetic map of 13,551 sequence-related amplified polymorphism markers (Sun *et al.* 2007), this map may facilitate fine mapping, gene cloning, global association mapping of cotton genes and traits, and other genomic studies.

### Distribution of markers

Genetic maps always face the problem of unevenly distributed markers and the resulting gaps. During meiosis, recombination does not happen evenly on the chromosomes. Further, marker sequences are not randomly dispersed, especially for sequence repeats such as SSRs. In centromeric and intergenic regions, more sequence repeats were found (Paterson *et al.* 2012). Previous genetic maps illustrated the uneven distribution of markers with many large gaps; on a recombinational scale, distal chromosomal regions tended to have lower marker density than the centromere regions (Rong *et al.* 2004). On the physical scale used in this study, the distal gene-rich ends of the chromosomes clearly have greater marker density, a natural outcome of the fact that many of the markers used are gene-derived. Not a single 1-Mb window in any chromosome had fewer than nine markers (*i.e.*, an average of one per 110 kb), and most had at least 50 markers, guaranteeing the availability of established DNA markers in any region of interest.

### Alignment of QTL and RGA hotspots

Global genotyping is the trend for dissecting genes/QTL controlling important phenotypes, and prior QTL information provides valuable evidence toward validation of statistically significant associations from genome-wide association studies. The cotton community has identified at least hundreds of QTL for fiber-related traits, plant architecture, disease resistance, and stress tolerance [a meta-analysis now 6 years old already identified 432 QTL (Rong *et al.* 2007)]. Here, we provide resources to quickly and efficiently target QTL “hotspots” for fiber-related or disease/pest-related traits for rapid characterization, for example in searches for novel alleles. For example, genotyping of as few as 126 DNA markers would permit one to assess cosegregation at DNA markers flanking with 53% (159/300) of the RGAs in the cotton reference sequence.

### Genome variations revealed by comparative analysis of maps

Although the various cotton genomes have a high degree of colinearity, several rearrangements known from prior studies (Rong *et al.* 2004) and the five possible inversions identified here need to be accounted for in “translation” of information from the D-genome based WGMM to other cotton genomes. The At genome experienced two reciprocal chromosome arm translocations, between chromosome 02 and 03 and between chromosome 04 and 05, with break points near the centromere regions. These genome variations may facilitate the understanding of genome evolution and gene/QTL cloning from the related orthologous regions. Furthermore, to characterize the variations, the WGMM provides a foundation for and will facilitate the investigation of the relationships of phenotype and genotype of important agronomic traits, especially those controlled by A and At genomes. We noted that some of the newly identified and relatively smaller inversions could represent misassemblies of the reference genome sequence.

In conclusion, the consensus high-density WGMM is a valuable resource with the potential for adding additional value as more information accumulates, such as better knowledge of QTL hotspots for cotton fiber development, roles of different RGA clusters in conferring pest resistance genes and QTL, global association studies of cotton, and/or genome structure and variation.

## Supplementary Material

Supporting Information

Corrigendum
