# Supplementary material for: A Whole-Genome DNA Marker Map for Cotton Based on the D-Genome Sequence of Gossypium raimondii L
Source: G3 (Bethesda). 2013 Oct 1;3(10):1759–67. doi: 10.1534/g3.113.006890 (PMC3789800; doi:10.1534/g3.113.006890)
Supplement: Supporting Information [file supp_3_10_1759__index.html]

A Whole-Genome DNA Marker Map for Cotton Based on the D-Genome Sequence of Gossypium raimondii L. — Supporting Information 

# A Whole-Genome DNA Marker Map for Cotton Based on the D-Genome Sequence of *Gossypium raimondii* L.

## Supporting Information for Wang *et al.*, 2013

**Files in this Data Supplement:**

- Supporting Information - Tables S1-S4 (PDF, 578 KB)
- Table S2 - Marker density on the chromosomes of cotton D-genome pseudo molecules (PDF, 307 KB)
- Table S3 - Marker alignment of Di, At, Dt and consensus genetic maps to the D genome pseudo molecules (PDF, 346 KB)
- Table S4 - RGA clusters and their flanking markers on the D genome and the WGMM (PDF, 318 KB)
- Table S1 - Cotton WGMM based on the D genome sequence (.xlsx, 2 MB)
