# Supplementary material for: A Whole-Genome DNA Marker Map for Cotton Based on the D-Genome Sequence of Gossypium raimondii L
Source: G3 (Bethesda). 2013 Oct 1;3(10):1759–67. doi: 10.1534/g3.113.006890 (PMC3789800; doi:10.1534/g3.113.006890)
Supplement: Supporting Information [file supp_g3.113.006890_006890SI.pdf]

G3 · Genes | Genomes | Genetics

A Whole Genome DNA Marker Map Based on the D-genome Sequence of *Gossypium raimondii* L.

Zining Wang[1], Dong Zhang[1], Xiyin Wang, Xu Tan, Hui Guo, Andrew H. Paterson[2]

Plant Genome Mapping Laboratory, University of Georgia, 111 Riverbend Road, Athens GA, 30605

[1] These authors contributed to this work equally.

[2]Corresponding author: Plant Genome Mapping Laboratory (Dept #398), University of Georgia, 111 Riverbend Road, Rm 228, Athens GA 30605, Phone 1-706-583-0162, Fax 1-706-583-0160, Email paterson@plantbio.uga.edu

DOI: 10.1534/g3.113.006890

Table S1 Cotton WGMM based on the D genome sequence

Table S1 is available for download at http://www.g3journal.org/lookup/suppl/doi:10.1534/g3.113.006890/-/DC1.

Table S2 Marker density on the chromosomes of cotton D-genome pseudo molecules

D Pseudo Chr.	Chr. Length (bp)	Consensus Chr.	Consensus (cM)	No. of Markers	Density (kb/marker)	Di Chr.	At Chr.	Dt Chr.	References*
D01	54814742	C01	195.4	3724	14.7	Di01	Chr.07	Chr.16	Lacape et al. 2003
D02	62749324	C02	176.4	3719	16.9	Di02	Chr.01	Chr.15	Reinisch et al. 1994
D03	45751563	C03	120.7	2691	17	Di03	Chr.02,03	Chr.17	Rong et al. 2004
D04	62096951	C04	183.8	4077	15.2	Di04	LGA02	LGD03	Reinisch et al. 1994
D05	64063734	C05	191.9	3722	17.2	Di05	Chr.02,03	Chr.14	Rong et al. 2004
D06	51025885	C06	172.6	3372	15.1	Di06	Chr.09	Chr.23	Reinisch et al. 1994
D07	60741731	C07	217.2	4739	12.8	Di07	LGA03	LGD02	Reinisch et al. 1994
D08	57079257	C08	159.5	3995	14.3	Di08	Chr.12	Chr.26	Lacape et al. 2003
D09	70659608	C09	264.6	5370	13.2	Di09	Chr.04,05	LGD08	Rong et al. 2004
D10	62132915	C10	172.6	3357	18.5	Di10	Chr.06	Chr.25	Reinisch et al. 1994
D11	62588852	C11	170.2	3643	17.2	Di11	Chr.10	Chr.20	Lacape et al. 2003
D12	34941032	C12	101.7	2386	14.6	Di12	Chr.04,05	Chr.22	Rong et al. 2004
D13	58256473	C13	198.1	3752	15.5	Di13	LGA01	Chr.18	Lacape et al. 2003
Total	746902067		2325	48547	15.6				

*The consensus relationship of D, At and Dt subgenomes was based on Rong et al. 2005a.

Table S3 Marker alignment of Di, At, Dt and consensus genetic maps to the D genome pseudo molecules

	D1	D2	D3	D4	D5	D6	D7	D8	D9	D10	D11	D12	D13	Total
Di01	37	1	2	2	4	2	4	2	5	2	2		4	67
Di02	3	42		4	3	2	3	1	4	1	3	6	5	77
Di03	2	1	25	2		1	5	1	4		3	0	3	47
Di04	2		1	34	1	4	1	4	6	1	1	3	2	60
Di05	3	3	4	1	41	3	2	6	5	1	9	5	7	90
Di06	4	1	1	1	4	44		2	2			2	2	63
Di07	7		4	2		2	65	2	3		4	0		89
Di08	1	1	2	5	1	3	7	43	2	1	1	1	3	71
Di09	5	2	3	1	3	4	2	3	61	4	4	6	5	103
Di10	2					1		1	4	21			5	34
Di11	1		1	2	4	3	2	2	6	4	50	4	4	83
Di12	4	3		1	4	3	2	2	1	2	5	31	6	64
Di13	10		3	3	5	4	5	3	4		4	7	41	89
Total	81	54	46	58	70	76	98	72	107	37	86	65	87	937
Chr.07	58	1		1	4	2	7	7	9	4	2	3	5	103
Chr.01		52	1		1		1	2	2	1	1	1	3	65
Chr.02	1	4	20		24		2		1	1	4	1	5	63
L.G.A02	4	2	1	61	2	1	4	2	4	1		1	7	90
Chr.03	2	3	20	2	36	5	3	4	6		1	1	5	88
Chr.09	3	3	3	2	2	60		6	2	3	4	3	2	93
L.G.A03					1	2	71	3	4	2	3	1	2	89
Chr.12	2	2	2	4	2	2	4	80	3	1	1	1	2	106
Chr.04	5	7		2	3	2	8	7	25	3	1	25	4	92
Chr.06	1	2		1			2	2	5	41			3	57
Chr.10	1	1	2	5	2	2	6	4	2	4	60	3	2	94
Chr.05	2			4	3	1	1	1	93		2	25	4	136
L.G.A01	2	3	1	1	3	1	3	2	1	7		3	67	94
Total	81	80	50	83	83	78	112	120	157	68	79	68	111	1170
Chr.16	52	3	1	2	3	1	4	1	3	3	1	1	5	80
Chr.15	4	65	3	3	3	1		2	7	2	3	1	3	97
Chr.17	2	1	35	2	3	2	2	1		1	1	1	3	54
L.G.D03	3	3	4	66	3	1	4	1	3		2	3	3	96
Chr.14	2	4		3	70		2		5		4	4	2	96
Chr.23	4		3	3	1	53	4	2	6			7	1	84
L.G.D02	4	6	3	2	3	4	79	2	9	4	5	5	9	135
Chr.26	3	4	1	4	4	3	2	64	1	1	1	1	4	93
L.G.D08	4	2	2	4	1	3	3	1	95	2	5		2	124
Chr.25	3	4	1	1	1	1	4	1	6	52	3		3	80
Chr.20	1	2	1	1	5	1	4	1		2	66	2	2	88
Chr.22	5	5	2	1	3	4	1	4	6	3	1	38	3	76
Chr.18	4	5	1	3	5		4	6	3	3	2	1	66	103
Total	91	104	57	95	105	74	113	86	144	73	94	64	106	1206
C01	117	5	5	8	7	8	8	4	10	5	4	8	9	198
C02	8	122	3	8	12	5	9	8	11	7	6	13	12	224

C03	3	4	86	6	6	7	7	4	6	1	6	2	5	143
C04	5	3	3	125	5	7	5	12	12	3	5	6	6	197
C05	9	7	7	4	131	6	5	11	9	2	12	12	15	230
C06	7	2	5	3	9	137	6	6	6	1	3	6	3	194
C07	16	1	7	10	6	6	174	8	11	6	11	4	7	267
C08	8	4	3	8	5	10	12	153	8	4	6	8	10	239
C09	17	11	6	6	11	9	13	7	220	14	6	27	7	354
C10	8	3	1	1	1	4	4	3	7	92	6	3	14	147
C11	4	4	4	4	12	7	10	4	12	6	145	6	6	224
C12	8	5	1	5	9	11	8	3	4	2	10	86	10	162
C13	20	7	11	11	11	9	13	5	9	5	8	12	137	258
Total	230	178	142	199	225	226	274	228	325	148	228	193	241	2837

Table S4 RGA clusters and their flanking markers on the D genome and the WGMM

Chr.	Start bp	End bp	Flanking Markers	Genes
D02	1259105	1294678	PVNC035;MON_DPL0398	Gorai.002G018500;Gorai.002G018600;Gorai.002G018700;Gorai.002G018800;<br>Gorai.002G018900;Gorai.002G019000
D02	3676992	3687083	HAU0956;NAU3927	Gorai.002G044500;Gorai.002G044600
D02	3706720	3757607	HAU0956;NAU3927	Gorai.002G044800;Gorai.002G044900;Gorai.002G045000
D02	3917161	3944157	BNL2664;HAU2909	Gorai.002G046500;Gorai.002G046600
D03	43764797	43807236	Gafb24D05;BNL2690	Gorai.003G167500;Gorai.003G167600
D03	44353765	44368552	MUSB198;MUSB198	Gorai.003G172600;Gorai.003G172700
D03	44432530	44453739	MUSB198;MUSB198	Gorai.003G173100;Gorai.003G173200
D03	44496441	44516569	Gh376;STV026	Gorai.003G173800;Gorai.003G173900
D03	44571575	44594330	DOW062;pAR0607	Gorai.003G174400;Gorai.003G174500
D04	46741939	46752567	MON_CGR5552;MON_CGR6762	Gorai.004G169300;Gorai.004G169400
D05	661191	679262	MON_CGR5534;HAU2197	Gorai.005G009600;Gorai.005G009700
D05	922957	932420	MON_DPL0682;Gate1AF08a	Gorai.005G012600;Gorai.005G012700
D05	54922608	54956023	CIR140;PAR0850	Gorai.005G188500;Gorai.005G188600
D07	16647870	16676788	HAU1699;Gh606	Gorai.007G178900;Gorai.007G179000
D07	49637952	49779990	DOW019;MUSB1005	Gorai.007G290200;Gorai.007G290300;Gorai.007G290400
D07	49884673	49917741	BNL1017;MUSB1005	Gorai.007G290900;Gorai.007G291000
D07	53339307	53377278	Gh132;MON_CGR5232	Gorai.007G318800;Gorai.007G318900
D07	53522590	53530374	MUSB296;pAR03D06	Gorai.007G319800;Gorai.007G319900
D07	53645114	53668300	pAR03D06;Unig22B08	Gorai.007G320900;Gorai.007G321000;Gorai.007G321100
D07	53712642	53768343	Unig22B08;MUSB261	Gorai.007G321700;Gorai.007G321800
D07	54264835	54348738	NBRI_Gh_K029;TMB2061	Gorai.007G324100;Gorai.007G324200;Gorai.007G324300;Gorai.007G324400
D07	54394357	54408928	TMB1871;pGH650	Gorai.007G324600;Gorai.007G324700
D07	55343090	55387014	TMB1434;BNL3569	Gorai.007G330900;Gorai.007G331000
D07	55411056	55446114	MON_DPL0289;BNL3569	Gorai.007G331400;Gorai.007G331500;Gorai.007G331600
D07	55600682	55625706	NBRI_Gh_A_EYI1BW401A0QPI;MUSB291	Gorai.007G333200;Gorai.007G333300
D07	55670576	55704436	MON_CGR6558;MON_DPL0372	Gorai.007G333700;Gorai.007G333800;Gorai.007G333900
D07	58647540	58719479	STV125;NBS008	Gorai.007G356000;Gorai.007G356100

D07	58785596	58835258	pAR0003;NBS008	Gorai.007G356800;Gorai.007G356900
D07	58960505	58968563	MUSB1236;HAU1054	Gorai.007G357700;Gorai.007G357800
D07	59223291	59325072	MON_C2;0120;MUSB1236	Gorai.007G361000;Gorai.007G361100;Gorai.007G361200;Gorai.007G361300;Gorai.007G361400;
D07	59599967	59637559	A1547;A1547	Gorai.007G364300;Gorai.007G364400
D07	59703598	59723966	NAU871;NBRI_Gh_E016	Gorai.007G364900;Gorai.007G365000
D07	59745456	59769095	MUSB517;NBRI_Gh_C101	Gorai.007G365300;Gorai.007G365400;Gorai.007G365500
D07	59802082	59832604	MON_DPL0260;NBRI_Gh_F029F	Gorai.007G365700;Gorai.007G365800;Gorai.007G365900
D07	59885118	59924863	MON_DPL0289;MON_DPL0289	Gorai.007G366100;Gorai.007G366200
D09	588589	607264	GA__Ea0004N11;NBRI_Gh_PD_77	Gorai.009G007000;Gorai.009G007100;Gorai.009G007200
D09	29098195	29109037	NAU4011;MON_SHIN;1099	Gorai.009G311700;Gorai.009G311800
D09	29725206	29748568	NBRI_Gh_PD_23;HAU2059	Gorai.009G315400;Gorai.009G315500
D09	50542604	50589086	NAU3549;NBRI_Gh_C_EYT27PB01CEZ1A	Gorai.009G373100;Gorai.009G373200
D09	50649534	50717845	NBRI_Gh_C_EYT27PB01CEZ1A; NBRI_Gh_E_EYT27PB03GDPRM	Gorai.009G373600;Gorai.009G373700;Gorai.009G373800;Gorai.009G373900
D09	50766064	50827552	HAU1382;NBRI_Gh_E_EYT27PB03GDPRM	Gorai.009G374200;Gorai.009G374300
D09	51519828	51639095	MON_DPL0271;MUSB410	Gorai.009G379600;Gorai.009G379700;Gorai.009G379800;Gorai.009G379900;Gorai.009G380000; Gorai.009G380100;Gorai.009G380200
D09	51637604	51693368	MON_DPL0271;MUSB410	Gorai.009G380400;Gorai.009G380500;Gorai.009G380600;Gorai.009G380700
D09	51785004	51800282	BNL3858;MUSB409	Gorai.009G381300;Gorai.009G381400;Gorai.009G381500
D09	52409587	52471137	MON_DPL0271;TMB1203	Gorai.009G386400;Gorai.009G386500;Gorai.009G386600;Gorai.009G386700
D09	52543267	52559033	TMB1203;NBRI_Gh_B_EYI1BW404IOOKX	Gorai.009G387500;Gorai.009G387600
D09	68627692	68710879	TMB283;NBRI_Gh_D_2079	Gorai.009G436400;Gorai.009G436500;Gorai.009G436600;Gorai.009G436700
D09	68721989	68752363	MON_DPL0776;MUSB264	Gorai.009G436900;Gorai.009G437000
D09	69307193	69323105	NAU1080;MUSB928	Gorai.009G441400;Gorai.009G441500
D10	2672424	2680912	Gate2AC02b;MON_DPL0465	Gorai.010G031100;Gorai.010G031200
D10	59682531	59688804	NAU4963;JESPR265	Gorai.010G224500;Gorai.010G224600
D10	59756229	59773609	NAU4963;JESPR265	Gorai.010G225700;Gorai.010G225800
D10	59805063	59826579	NAU4963;JESPR265	Gorai.010G226700;Gorai.010G226800
D10	60107252	60120427	MON_DPL0918;MON_DPL0918	Gorai.010G229400;Gorai.010G229500
D10	60426658	60435931	Gh100;Gh350	Gorai.010G233400;Gorai.010G233500
D11	20203277	20229559	Unig24E01;Coau107	Gorai.011G133300;Gorai.011G133400

D11	55881392	55886994	Gh176;TMB1497	Gorai.011G237700;Gorai.011G237800
D11	59369171	59395127	MON_DC40188;MUSB989	Gorai.011G263000;Gorai.011G263100
D11	60168653	60204227	Gate4AF02;G1237	Gorai.011G270000;Gorai.011G270100
D11	61230953	61265634	G1104;MUSB1048	Gorai.011G280600;Gorai.011G280700
D11	61373445	61430324	G1261k;Unig26B07	Gorai.011G281900;Gorai.011G282000;Gorai.011G282100;Gorai.011G282200
D11	61465710	61488545	NBRI_Gh_C_EYT27PB01BIDYK;HAU3308	Gorai.011G282400;Gorai.011G282500
D13	55041135	55093728	NBRI_Gh_C_EYT27PB01BOU6H;Gh181	Gorai.013G232000;Gorai.013G232100;Gorai.013G232200;Gorai.013G232300

*Each row is a cluster.
