# Supplementary material for: A Whole-Genome DNA Marker Map for Cotton Based on the D-Genome Sequence of Gossypium raimondii L
Source: G3 (Bethesda). 2013 Oct 1;3(10):1759–67. doi: 10.1534/g3.113.006890 (PMC3789800; doi:10.1534/g3.113.006890)
Supplement: Supporting Information [file supp_g3.113.006890_TableS2.pdf]

Table S2 Marker density on the chromosomes of cotton D-genome pseudo molecules

D Pseudo Chr.	Chr. Length (bp)	Consensus Chr.	Consensus (cM)	No. of Markers	Density (kb/marker)	Di Chr.	At Chr.	Dt Chr.	References*
D01	54814742	C01	195.4	3724	14.7	Di01	Chr.07	Chr.16	Lacape et al. 2003
D02	62749324	C02	176.4	3719	16.9	Di02	Chr.01	Chr.15	Reinisch et al. 1994
D03	45751563	C03	120.7	2691	17	Di03	Chr.02,03	Chr.17	Rong et al. 2004
D04	62096951	C04	183.8	4077	15.2	Di04	LGA02	LGD03	Reinisch et al. 1994
D05	64063734	C05	191.9	3722	17.2	Di05	Chr.02,03	Chr.14	Rong et al. 2004
D06	51025885	C06	172.6	3372	15.1	Di06	Chr.09	Chr.23	Reinisch et al. 1994
D07	60741731	C07	217.2	4739	12.8	Di07	LGA03	LGD02	Reinisch et al. 1994
D08	57079257	C08	159.5	3995	14.3	Di08	Chr.12	Chr.26	Lacape et al. 2003
D09	70659608	C09	264.6	5370	13.2	Di09	Chr.04,05	LGD08	Rong et al. 2004
D10	62132915	C10	172.6	3357	18.5	Di10	Chr.06	Chr.25	Reinisch et al. 1994
D11	62588852	C11	170.2	3643	17.2	Di11	Chr.10	Chr.20	Lacape et al. 2003
D12	34941032	C12	101.7	2386	14.6	Di12	Chr.04,05	Chr.22	Rong et al. 2004
D13	58256473	C13	198.1	3752	15.5	Di13	LGA01	Chr.18	Lacape et al. 2003
Total	746902067		2325	48547	15.6				

*The consensus relationship of D, At and Dt subgenomes was based on Rong et al. 2005a.
