# Supplementary material for: A Whole-Genome DNA Marker Map for Cotton Based on the D-Genome Sequence of Gossypium raimondii L
Source: G3 (Bethesda). 2013 Oct 1;3(10):1759–67. doi: 10.1534/g3.113.006890 (PMC3789800; doi:10.1534/g3.113.006890)
Supplement: Supporting Information [file supp_g3.113.006890_TableS3.pdf]

Table S3 Marker alignment of Di, At, Dt and consensus genetic maps to the D genome pseudo molecules

	D1	D2	D3	D4	D5	D6	D7	D8	D9	D10	D11	D12	D13	Total
Di01	37	1	2	2	4	2	4	2	5	2	2		4	67
Di02	3	42		4	3	2	3	1	4	1	3	6	5	77
Di03	2	1	25	2		1	5	1	4		3	0	3	47
Di04	2		1	34	1	4	1	4	6	1	1	3	2	60
Di05	3	3	4	1	41	3	2	6	5	1	9	5	7	90
Di06	4	1	1	1	4	44		2	2			2	2	63
Di07	7		4	2		2	65	2	3		4	0		89
Di08	1	1	2	5	1	3	7	43	2	1	1	1	3	71
Di09	5	2	3	1	3	4	2	3	61	4	4	6	5	103
Di10	2					1		1	4	21			5	34
Di11	1		1	2	4	3	2	2	6	4	50	4	4	83
Di12	4	3		1	4	3	2	2	1	2	5	31	6	64
Di13	10		3	3	5	4	5	3	4		4	7	41	89
Total	81	54	46	58	70	76	98	72	107	37	86	65	87	937
Chr.07	58	1		1	4	2	7	7	9	4	2	3	5	103
Chr.01		52	1		1		1	2	2	1	1	1	3	65
Chr.02	1	4	20		24		2		1	1	4	1	5	63
L.G.A02	4	2	1	61	2	1	4	2	4	1		1	7	90
Chr.03	2	3	20	2	36	5	3	4	6		1	1	5	88
Chr.09	3	3	3	2	2	60		6	2	3	4	3	2	93
L.G.A03					1	2	71	3	4	2	3	1	2	89
Chr.12	2	2	2	4	2	2	4	80	3	1	1	1	2	106
Chr.04	5	7		2	3	2	8	7	25	3	1	25	4	92
Chr.06	1	2		1			2	2	5	41			3	57
Chr.10	1	1	2	5	2	2	6	4	2	4	60	3	2	94
Chr.05	2			4	3	1	1	1	93		2	25	4	136
L.G.A01	2	3	1	1	3	1	3	2	1	7		3	67	94
Total	81	80	50	83	83	78	112	120	157	68	79	68	111	1170
Chr.16	52	3	1	2	3	1	4	1	3	3	1	1	5	80
Chr.15	4	65	3	3	3	1		2	7	2	3	1	3	97
Chr.17	2	1	35	2	3	2	2	1		1	1	1	3	54
L.G.D03	3	3	4	66	3	1	4	1	3		2	3	3	96
Chr.14	2	4		3	70		2		5		4	4	2	96
Chr.23	4		3	3	1	53	4	2	6			7	1	84
L.G.D02	4	6	3	2	3	4	79	2	9	4	5	5	9	135
Chr.26	3	4	1	4	4	3	2	64	1	1	1	1	4	93
L.G.D08	4	2	2	4	1	3	3	1	95	2	5		2	124
Chr.25	3	4	1	1	1	1	4	1	6	52	3		3	80
Chr.20	1	2	1	1	5	1	4	1		2	66	2	2	88
Chr.22	5	5	2	1	3	4	1	4	6	3	1	38	3	76
Chr.18	4	5	1	3	5		4	6	3	3	2	1	66	103
Total	91	104	57	95	105	74	113	86	144	73	94	64	106	1206
C01	117	5	5	8	7	8	8	4	10	5	4	8	9	198
C02	8	122	3	8	12	5	9	8	11	7	6	13	12	224

C03	3	4	86	6	6	7	7	4	6	1	6	2	5	143
C04	5	3	3	125	5	7	5	12	12	3	5	6	6	197
C05	9	7	7	4	131	6	5	11	9	2	12	12	15	230
C06	7	2	5	3	9	137	6	6	6	1	3	6	3	194
C07	16	1	7	10	6	6	174	8	11	6	11	4	7	267
C08	8	4	3	8	5	10	12	153	8	4	6	8	10	239
C09	17	11	6	6	11	9	13	7	220	14	6	27	7	354
C10	8	3	1	1	1	4	4	3	7	92	6	3	14	147
C11	4	4	4	4	12	7	10	4	12	6	145	6	6	224
C12	8	5	1	5	9	11	8	3	4	2	10	86	10	162
C13	20	7	11	11	11	9	13	5	9	5	8	12	137	258
Total	230	178	142	199	225	226	274	228	325	148	228	193	241	2837
