# Supplementary material for: A Whole-Genome DNA Marker Map for Cotton Based on the D-Genome Sequence of Gossypium raimondii L
Source: G3 (Bethesda). 2013 Oct 1;3(10):1759–67. doi: 10.1534/g3.113.006890 (PMC3789800; doi:10.1534/g3.113.006890)
Supplement: Supporting Information [file supp_g3.113.006890_TableS4.pdf]

Table S4 RGA clusters and their flanking markers on the D genome and the WGMM

Chr.	Start bp	End bp	Flanking Markers	Genes
D02	1259105	1294678	PVNC035;MON_DPL0398	Gorai.002G018500;Gorai.002G018600;Gorai.002G018700;Gorai.002G018800; Gorai.002G018900;Gorai.002G019000
D02	3676992	3687083	HAU0956;NAU3927	Gorai.002G044500;Gorai.002G044600
D02	3706720	3757607	HAU0956;NAU3927	Gorai.002G044800;Gorai.002G044900;Gorai.002G045000
D02	3917161	3944157	BNL2664;HAU2909	Gorai.002G046500;Gorai.002G046600
D03	43764797	43807236	Gafb24D05;BNL2690	Gorai.003G167500;Gorai.003G167600
D03	44353765	44368552	MUSB198;MUSB198	Gorai.003G172600;Gorai.003G172700
D03	44432530	44453739	MUSB198;MUSB198	Gorai.003G173100;Gorai.003G173200
D03	44496441	44516569	Gh376;STV026	Gorai.003G173800;Gorai.003G173900
D03	44571575	44594330	DOW062;pAR0607	Gorai.003G174400;Gorai.003G174500
D04	46741939	46752567	MON_CGR5552;MON_CGR6762	Gorai.004G169300;Gorai.004G169400
D05	661191	679262	MON_CGR5534;HAU2197	Gorai.005G009600;Gorai.005G009700
D05	922957	932420	MON_DPL0682;Gate1AF08a	Gorai.005G012600;Gorai.005G012700
D05	54922608	54956023	CIR140;PAR0850	Gorai.005G188500;Gorai.005G188600
D07	16647870	16676788	HAU1699;Gh606	Gorai.007G178900;Gorai.007G179000
D07	49637952	49779990	DOW019;MUSB1005	Gorai.007G290200;Gorai.007G290300;Gorai.007G290400
D07	49884673	49917741	BNL1017;MUSB1005	Gorai.007G290900;Gorai.007G291000
D07	53339307	53377278	Gh132;MON_CGR5232	Gorai.007G318800;Gorai.007G318900
D07	53522590	53530374	MUSB296;pAR03D06	Gorai.007G319800;Gorai.007G319900
D07	53645114	53668300	pAR03D06;Unig22B08	Gorai.007G320900;Gorai.007G321000;Gorai.007G321100
D07	53712642	53768343	Unig22B08;MUSB261	Gorai.007G321700;Gorai.007G321800
D07	54264835	54348738	NBRI_Gh_K029;TMB2061	Gorai.007G324100;Gorai.007G324200;Gorai.007G324300;Gorai.007G324400
D07	54394357	54408928	TMB1871;pGH650	Gorai.007G324600;Gorai.007G324700
D07	55343090	55387014	TMB1434;BNL3569	Gorai.007G330900;Gorai.007G331000
D07	55411056	55446114	MON_DPL0289;BNL3569	Gorai.007G331400;Gorai.007G331500;Gorai.007G331600
D07	55600682	55625706	NBRI_Gh_A_EYI1BW401A0QPI;MUSB291	Gorai.007G333200;Gorai.007G333300
D07	55670576	55704436	MON_CGR6558;MON_DPL0372	Gorai.007G333700;Gorai.007G333800;Gorai.007G333900
D07	58647540	58719479	STV125;NBS008	Gorai.007G356000;Gorai.007G356100

D07	58785596	58835258	pAR0003;NBS008	Gorai.007G356800;Gorai.007G356900
D07	58960505	58968563	MUSB1236;HAU1054	Gorai.007G357700;Gorai.007G357800
D07	59223291	59325072	MON_C2;0120;MUSB1236	Gorai.007G361000;Gorai.007G361100;Gorai.007G361200;Gorai.007G361300;Gorai.007G361400;
D07	59599967	59637559	A1547;A1547	Gorai.007G364300;Gorai.007G364400
D07	59703598	59723966	NAU871;NBRI_Gh_E016	Gorai.007G364900;Gorai.007G365000
D07	59745456	59769095	MUSB517;NBRI_Gh_C101	Gorai.007G365300;Gorai.007G365400;Gorai.007G365500
D07	59802082	59832604	MON_DPL0260;NBRI_Gh_F029F	Gorai.007G365700;Gorai.007G365800;Gorai.007G365900
D07	59885118	59924863	MON_DPL0289;MON_DPL0289	Gorai.007G366100;Gorai.007G366200
D09	588589	607264	GA__Ea0004N11;NBRI_Gh_PD_77	Gorai.009G007000;Gorai.009G007100;Gorai.009G007200
D09	29098195	29109037	NAU4011;MON_SHIN;1099	Gorai.009G311700;Gorai.009G311800
D09	29725206	29748568	NBRI_Gh_PD_23;HAU2059	Gorai.009G315400;Gorai.009G315500
D09	50542604	50589086	NAU3549;NBRI_Gh_C_EYT27PB01CEZ1A	Gorai.009G373100;Gorai.009G373200
D09	50649534	50717845	NBRI_Gh_C_EYT27PB01CEZ1A; NBRI_Gh_E_EYT27PB03GDPRM	Gorai.009G373600;Gorai.009G373700;Gorai.009G373800;Gorai.009G373900
D09	50766064	50827552	HAU1382;NBRI_Gh_E_EYT27PB03GDPRM	Gorai.009G374200;Gorai.009G374300
D09	51519828	51639095	MON_DPL0271;MUSB410	Gorai.009G379600;Gorai.009G379700;Gorai.009G379800;Gorai.009G379900;Gorai.009G380000; Gorai.009G380100;Gorai.009G380200
D09	51637604	51693368	MON_DPL0271;MUSB410	Gorai.009G380400;Gorai.009G380500;Gorai.009G380600;Gorai.009G380700
D09	51785004	51800282	BNL3858;MUSB409	Gorai.009G381300;Gorai.009G381400;Gorai.009G381500
D09	52409587	52471137	MON_DPL0271;TMB1203	Gorai.009G386400;Gorai.009G386500;Gorai.009G386600;Gorai.009G386700
D09	52543267	52559033	TMB1203;NBRI_Gh_B_EYI1BW404IOOKX	Gorai.009G387500;Gorai.009G387600
D09	68627692	68710879	TMB283;NBRI_Gh_D_2079	Gorai.009G436400;Gorai.009G436500;Gorai.009G436600;Gorai.009G436700
D09	68721989	68752363	MON_DPL0776;MUSB264	Gorai.009G436900;Gorai.009G437000
D09	69307193	69323105	NAU1080;MUSB928	Gorai.009G441400;Gorai.009G441500
D10	2672424	2680912	Gate2AC02b;MON_DPL0465	Gorai.010G031100;Gorai.010G031200
D10	59682531	59688804	NAU4963;JESPR265	Gorai.010G224500;Gorai.010G224600
D10	59756229	59773609	NAU4963;JESPR265	Gorai.010G225700;Gorai.010G225800
D10	59805063	59826579	NAU4963;JESPR265	Gorai.010G226700;Gorai.010G226800
D10	60107252	60120427	MON_DPL0918;MON_DPL0918	Gorai.010G229400;Gorai.010G229500
D10	60426658	60435931	Gh100;Gh350	Gorai.010G233400;Gorai.010G233500
D11	20203277	20229559	Unig24E01;Coau107	Gorai.011G133300;Gorai.011G133400

D11	55881392	55886994	Gh176;TMB1497	Gorai.011G237700;Gorai.011G237800
D11	59369171	59395127	MON_DC40188;MUSB989	Gorai.011G263000;Gorai.011G263100
D11	60168653	60204227	Gate4AF02;G1237	Gorai.011G270000;Gorai.011G270100
D11	61230953	61265634	G1104;MUSB1048	Gorai.011G280600;Gorai.011G280700
D11	61373445	61430324	G1261k;Unig26B07	Gorai.011G281900;Gorai.011G282000;Gorai.011G282100;Gorai.011G282200
D11	61465710	61488545	NBRI_Gh_C_EYT27PB01BIDYK;HAU3308	Gorai.011G282400;Gorai.011G282500
D13	55041135	55093728	NBRI_Gh_C_EYT27PB01BOU6H;Gh181	Gorai.013G232000;Gorai.013G232100;Gorai.013G232200;Gorai.013G232300

*Each row is a cluster.
