# Supplementary material for: A Whole-Genome DNA Marker Map for Cotton Based on the D-Genome Sequence of Gossypium raimondii L
Source: G3 (Bethesda). 2013 Oct 1;3(10):1759–67. doi: 10.1534/g3.113.006890 (PMC3789800; doi:10.1534/g3.113.006890)
Supplement: Corrigendum [file supp_3_10_1759_v2_index.html]

Corrigendum 

# A Whole-Genome DNA Marker Map for Cotton Based on the D-Genome Sequence of *Gossypium raimondii* L.

## Corrigendum for Wang *et al.*, G3: Genes|Genomes|Genetics 3 (10) 1759-1767.

**Files in this Data Supplement:**

- Corrigendum - Corrigendum for Wang *et al.*, G3: Genes|Genomes|Genetics 3 (10) 1759-1767.
