# Supplementary material for: A Whole-Genome DNA Marker Map for Cotton Based on the D-Genome Sequence of Gossypium raimondii L
Source: G3 (Bethesda). 2013 Oct 1;3(10):1759–67. doi: 10.1534/g3.113.006890 (PMC3789800; doi:10.1534/g3.113.006890)
Supplement: Corrigendum [file supp_g3.113.006890_Corrigendum_Wang.pdf]

Corrigendum for Wang *et al.*, *G3: Genes|Genomes|Genetics* 3 (10) 1759-1767.

G3: Genes|Genomes|Genetics, Vol 3, 1759-1767, October 2013, Copyright © 2013 Genetics Society of America.

CORRIGENDUM

In the article by Z. Wang, D. Zhang, X. Wang, X. Tan, H. Guo, *et al.* (G3 3: 1759-1767) entitled "A Whole-Genome DNA Marker Map for Cotton Based on the D-Genome Sequence of *Gossypium raimondii* L.", Table 2 contained information in the third column (D-genome chromosome identities) that was not consistent with the information in the first column (tetraploid chromosome identities). Table 2 has now been corrected in the publication.
